# Risk of Abnormal Uterine Bleeding in Women with Chronic Spontaneous Urticaria: Korean Nationwide Population-Based Cohort

**DOI:** 10.3390/jcm15176807

**Published:** 2026-09-02

**Authors:** Su Yeon Park, Jee Yeon Kim, Ji-Eun Song

**Affiliations:** 1 Department of Obstetrics and Gynecology, Inha University College of Medicine, Inha University Hospital, Incheon 22332, Republic of Korea; sypark832@inha.ac.kr (S.Y.P.); jykim0901@inhauh.com (J.Y.K.); 2 Department of Obstetrics and Gynecology, Hallym University College of Medicine, Kangnam Sacred Heart Hospital, Seoul 07441, Republic of Korea

**Keywords:** abnormal uterine bleeding, chronic spontaneous urticaria, mast cell activation, sex hormones, hormonal dysregulation, nationwide population-based cohort

## Abstract

**Background**: Chronic spontaneous urticaria (CSU) is an immune-mediated disorder driven by mast cell activation, whereas abnormal uterine bleeding (AUB) is a heterogeneous condition with diverse etiologies. Although immune and inflammatory dysregulation has been independently implicated in CSU and certain forms of AUB, the association between CSU and AUB remains unclear. We therefore examined whether CSU was associated with AUB. **Methods**: We performed a retrospective population-based cohort analysis using data from the Korean National Health Insurance Service. Women newly diagnosed with CSU between 2009 and 2021 were individually matched 1:1 with controls according to age, income, and region of residence. Cox proportional hazards regression and Kaplan–Meier analysis were used to evaluate the risk of AUB. **Results**: The final cohort comprised 890,010 women with CSU and an equal number of matched controls. The incidence rates of AUB were 12.50 and 10.90 cases per 1000 person–years, respectively, yielding an absolute incidence rate difference of 1.60 cases per 1000 person–years (95% CI, 1.54–1.81). CSU was associated with an increased risk of AUB (aHR, 1.155; 95% CI, 1.142–1.168), and this association persisted among women aged <50 years. **Conclusions**: CSU was associated with a modestly increased risk of AUB, and this association persisted among women aged <50 years. These findings provide an initial epidemiological signal supporting further investigation, although the underlying mechanisms and associations with specific etiological subtypes of AUB remain unclear.

## 1. Introduction

Abnormal uterine bleeding (AUB), defined as abnormalities in menstrual frequency, regularity, duration, or volume, is common in reproductive-aged women [1,2]. According to the FIGO PALM-COEIN system, AUB has heterogeneous structural and non-structural etiologies, including polyps, adenomyosis, leiomyomas, malignancy and hyperplasia, coagulopathy, ovulatory dysfunction, endometrial disorders, iatrogenic causes, and causes not otherwise classified [1,2]. It affects approximately 3–30% of women of reproductive age, with up to one-third experiencing AUB at some point in their lifetime [2].

Chronic spontaneous urticaria (CSU) is an inflammatory skin disease that manifests as recurrent wheals or angioedema for longer than 6 weeks [3]. Although CSU affects approximately 0.5–1% of the general population, it occurs disproportionately in women of reproductive age, with the highest frequency of onset between 20 and 40 years [4].

Previous studies have suggested that female sex hormone imbalance, particularly involving estrogen and progesterone, can influence mast cell activation and histamine release, potentially triggering or exacerbating urticaria [5,6,7]. Kasperska-Zając et al. further described potential associations between the hormonal milieu and the clinical expression of urticaria during the menstrual cycle, pregnancy, menopause, and exposure to hormonal treatments [7]. Separately, immune and inflammatory processes have been implicated in certain forms of AUB. A recent Mendelian randomization study reported associations between specific immune-cell traits and selected AUB-related phenotypes, although these findings require clinical validation [8]. These observations provide biological plausibility for further investigation; however, direct evidence linking the two conditions through hormonal or inflammatory mechanisms remains scarce.

Accordingly, we performed a nationwide population-based cohort study using nationwide data from the South Korean National Health Insurance Service to evaluate the association between CSU and the subsequent development of AUB and to provide an epidemiological basis for future clinical and mechanistic investigations.

## 2. Materials and Methods

This study was approved by the Institutional Review Board of Inha University Hospital (approval code: 2025-07-022; approval date: 30 July 2025). The requirement for informed consent was waived by the Institutional Review Board because of the retrospective study design and the use of de-identified data. All study procedures were performed in accordance with the Declaration of Helsinki and applicable institutional guidelines. The South Korean National Health Insurance Database (NHID) has been described in detail elsewhere [9,10]. The NHID covers the Korean population and contains information on sociodemographic characteristics, health care utilization, birth and death dates, and national health-screening data. AUB and CSU were identified using claims-based operational definitions based on physician-assigned diagnostic codes. AUB was defined when at least one of the following International Classification of Diseases, 10th Revision (ICD-10) codes was recorded: N93.8, N93.801, N93.9, N92.601, N92.101, or N92.102. To reduce misclassification and ensure accurate diagnosis, cases with the following conditions were excluded: (1) Menopausal or postmenopausal bleeding: ICD-10 codes N92.4, N95.1, N95.9, N92.402, N95.8, N95.301, or N95.0. (2) Pregnancy- or miscarriage-related bleeding: ICD-10 codes Z34.80, Z34.81, Z34.82, or any code beginning with the letter “O” were excluded.

For the purposes of this claims-based study, CSU was operationally defined using the following physician-assigned ICD codes: L50.1, L50.2, L50.3, L50.4, L50.5, L50.6, L50.7, or L50.91. Because the NHID does not contain any detailed clinical symptoms, we could not directly verify the clinical criterion of recurrent wheals and/or angioedema lasting for more than 6 weeks. Accordingly, CSU in this study represents a claims-based diagnosis rather than a diagnosis confirmed through medical record review. To improve diagnostic specificity, we excluded patients before confirming CSU if they met either of the following criteria: (1) Urticaria with a specific underlying cause: Patients with urticaria defined by ICD codes 708.0, 708.2, 708.3, 708.4, or 708.5 were excluded; (2) Vasculitis or allergic purpura: Participants were excluded when vasculitis or allergic purpura was diagnosed within the 3-month period before or after the initial CSU diagnosis. This included ICD-9-CM code 287.0 and ICD-10 codes L95.8 (other vasculitis limited to the skin), L95.9 (vasculitis limited to the skin, unspecified), D69.0 (allergic purpura), and D69.006 (allergic vasculitis). Codes associated with vasculitis related to rheumatoid arthritis were also excluded: D89.107, M31.801, L95.0, M05.21, M05.22, M05.23, M05.24, M05.25, M05.27, M05.28, and M05.29.

Among the eligible women, 1,079,626 participants with CSU and 2,474,574 participants without CSU were identified between 2009 and 2021. To identify newly diagnosed CSU, 122,487 participants diagnosed with CSU in 2009 were excluded by applying 2009 as a washout period. The first qualifying CSU diagnosis was designated as the index date. An additional 67,129 participants with AUB before the index date were excluded, leaving 890,010 eligible participants with CSU.

**Figure 1:**
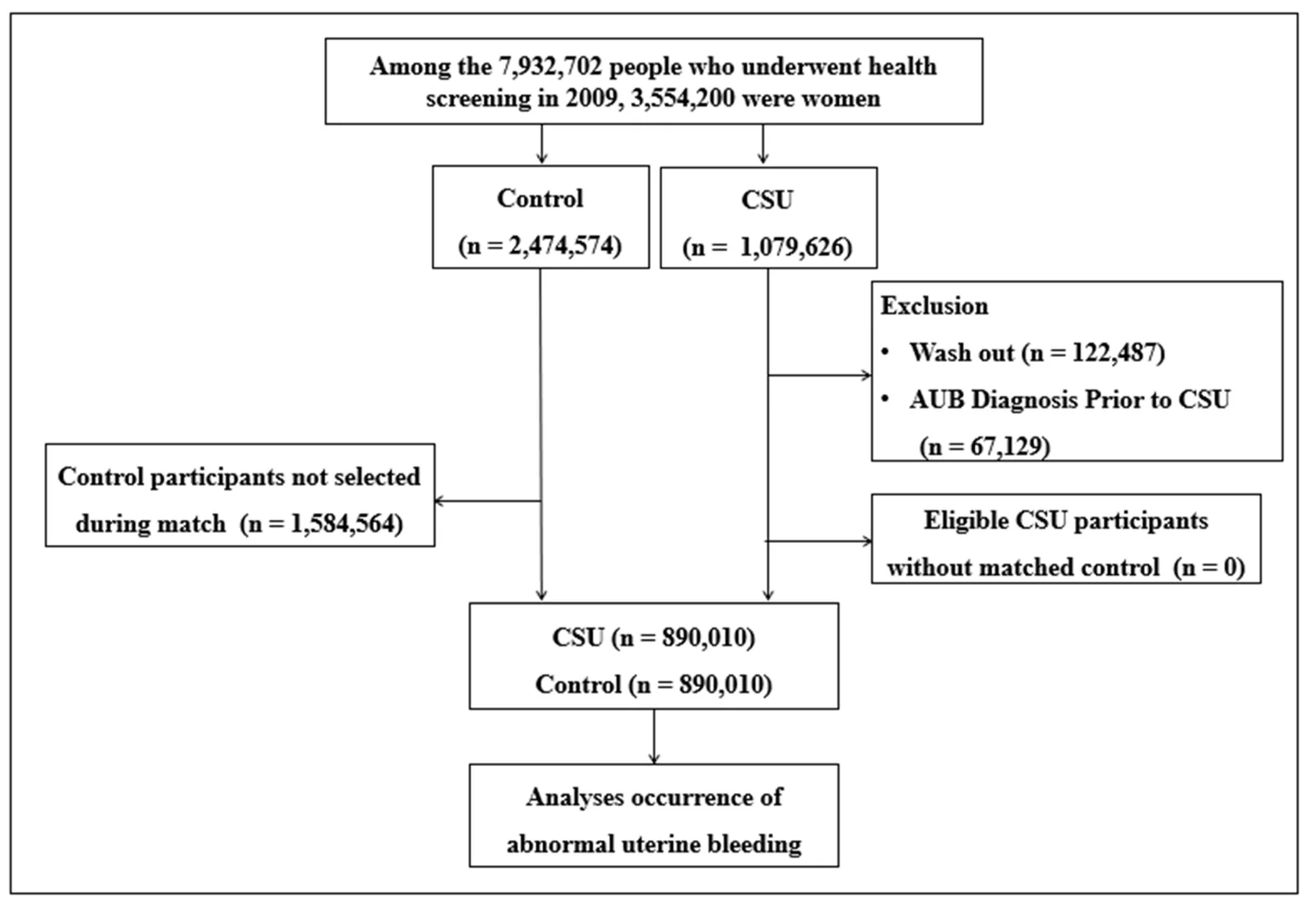
Flow diagram of participant selection. After matching for age, income, and region of residence, the final cohort comprised 890,010 participants with CSU and 890,010 controls.

Each eligible woman with CSU was matched with one control by exact matching according to age group, income category, and region of residence. Age was selected as a matching variable because the prevalence and clinical patterns of AUB vary across age groups [2]. Income and region of residence were included as proxies for socioeconomic status and geographical access to healthcare, respectively, because these factors may influence healthcare utilization and the likelihood of receiving a diagnosis of CSU or AUB. Within each matching stratum, eligible controls were randomly ordered and selected sequentially. Random ordering was used solely for control selection and did not constitute random allocation of participants to the study groups. Because this was a retrospective observational cohort study without an intervention, allocation concealment and participant or investigator blinding were not applicable. Each selected control was assigned the index date of the corresponding participant with CSU. Controls who had died or had been diagnosed with AUB before the assigned index date were considered ineligible. All eligible participants with CSU were successfully matched; therefore, no CSU participants remained unmatched. During this process, 1,584,564 control participants were either ineligible or not selected for the final matched cohort. Ultimately, 890,010 participants with CSU and 890,010 matched controls were included in the analysis (Figure 1).

Age was categorized into 13 groups (20–24, 25–29, …, ≥80 years). Income was divided into five levels, ranging from class 1 (lowest) to class 5 (highest), and region of residence was classified as urban or rural, consistent with our previous study [11].

Smoking, alcohol consumption, and obesity were included as potential lifestyle and metabolic confounders because previous epidemiological studies have reported associations between these factors and menstrual-cycle characteristics, including cycle irregularity and abnormal bleeding patterns [12,13]. Obesity was assessed using body mass index (kg/m^2^) and was included as a covariate in the adjusted model. Systolic blood pressure (SBP, mmHg), diastolic blood pressure (DBP, mmHg), and total cholesterol (mg/dL) were recorded as prespecified baseline cardiometabolic characteristics available from the national health screening database rather than as established AUB-specific risk factors. The Charlson Comorbidity Index (CCI) was included as a validated measure of overall comorbidity burden for risk adjustment in administrative health data [14]. CCI was analyzed as a continuous variable ranging from 0 (no comorbidities) to 29 (multiple comorbidities), with higher values indicating a greater comorbidity burden [14].

Baseline characteristics of the two groups were compared using standardized differences (Table 1). Stratified Cox regression was applied to estimate hazard ratios (HRs) and corresponding 95% confidence intervals (95% CIs) for incident AUB associated with CSU status. Follow-up began on the index date and continued until the first diagnosis of AUB, death, or the end of the study period, whichever occurred first. The crude model included CSU status as the exposure without additional covariates. The adjusted model included obesity, smoking, alcohol consumption, SBP, DBP, total cholesterol, and the CCI. Age, income, and region of residence, which were used for 1:1 matching, were specified as stratification variables to account for the matched cohort design. The stratified model allowed the baseline hazard to vary across strata while estimating a common HR for CSU relative to the control group.

Subgroup analyses were performed across categories of age, income, and region of residence using stratified Cox proportional hazards regression models. To evaluate the potential effect of outcome misclassification related to menopausal status, an age-restricted sensitivity analysis was performed among women aged <50 years, in whom postmenopausal bleeding was less likely. Incidence rates were obtained by dividing the number of AUB events by accumulated person–years and are reported per 1000 person–years. Absolute differences in incidence rates between the two groups were subsequently estimated. Differences in the cumulative probability of AUB were evaluated using Kaplan–Meier curves and log-rank tests. All tests were two-sided, with statistical significance defined as *p*-value <0.05. Statistical analyses were performed using SAS version 9.4 (SAS Institute Inc., Cary, NC, USA).

## 3. Results

Table 1 presents the baseline characteristics of the study population. Following 1:1 matching, each group contained 890,010 participants, yielding a total study population of 1,780,020. Age, income, and region of residence were identical between the groups, with a standardized difference of 0.000 for each variable. Standardized differences for the remaining covariates—including obesity, smoking, alcohol use, blood pressure, total cholesterol, and CCI—were all below 0.1, indicating minimal imbalance between the groups.

During the observation period, 65,417 women with CSU (7.35%) and 56,819 controls (6.38%) were newly diagnosed with AUB. The incidence rate was 12.50 per 1000 person–years in the CSU group and 10.90 per 1000 person–years in the control group, yielding an incidence rate difference of 1.60 per 1000 person–years (95% confidence interval [CI], 1.54–1.81). Compared with controls, participants with CSU had a crude HR of 1.154 (95% CI, 1.141–1.167) and an adjusted HR of 1.155 (95% CI, 1.142–1.168) for AUB (*p* < 0.001) (Table 2).

Potential associations between CSU and AUB across demographic subgroups were explored in analyses stratified by age, income, and region of residence (Table 2). These models incorporated obesity, smoking, alcohol use, blood pressure, total cholesterol, and CCI as adjustment variables. Participants aged <50 years had a slightly higher aHR (1.159; 95% CI, 1.145–1.174) than those aged ≥50 years (1.140; 95% CI, 1.111–1.171). Among low-income participants, the aHR was 1.161 (95% CI, 1.143–1.179), and among high-income participants, 1.149 (95% CI, 1.130–1.168). An increased risk was observed in both residential strata, with adjusted HRs of 1.167 (95% CI, 1.148–1.187) in urban residents and 1.145 (95% CI, 1.128–1.163) in rural residents, with aHRs of 1.167 (95% CI, 1.148–1.187) and 1.145 (95% CI, 1.128–1.163), respectively (all *p* < 0.001).

**Figure 2:**
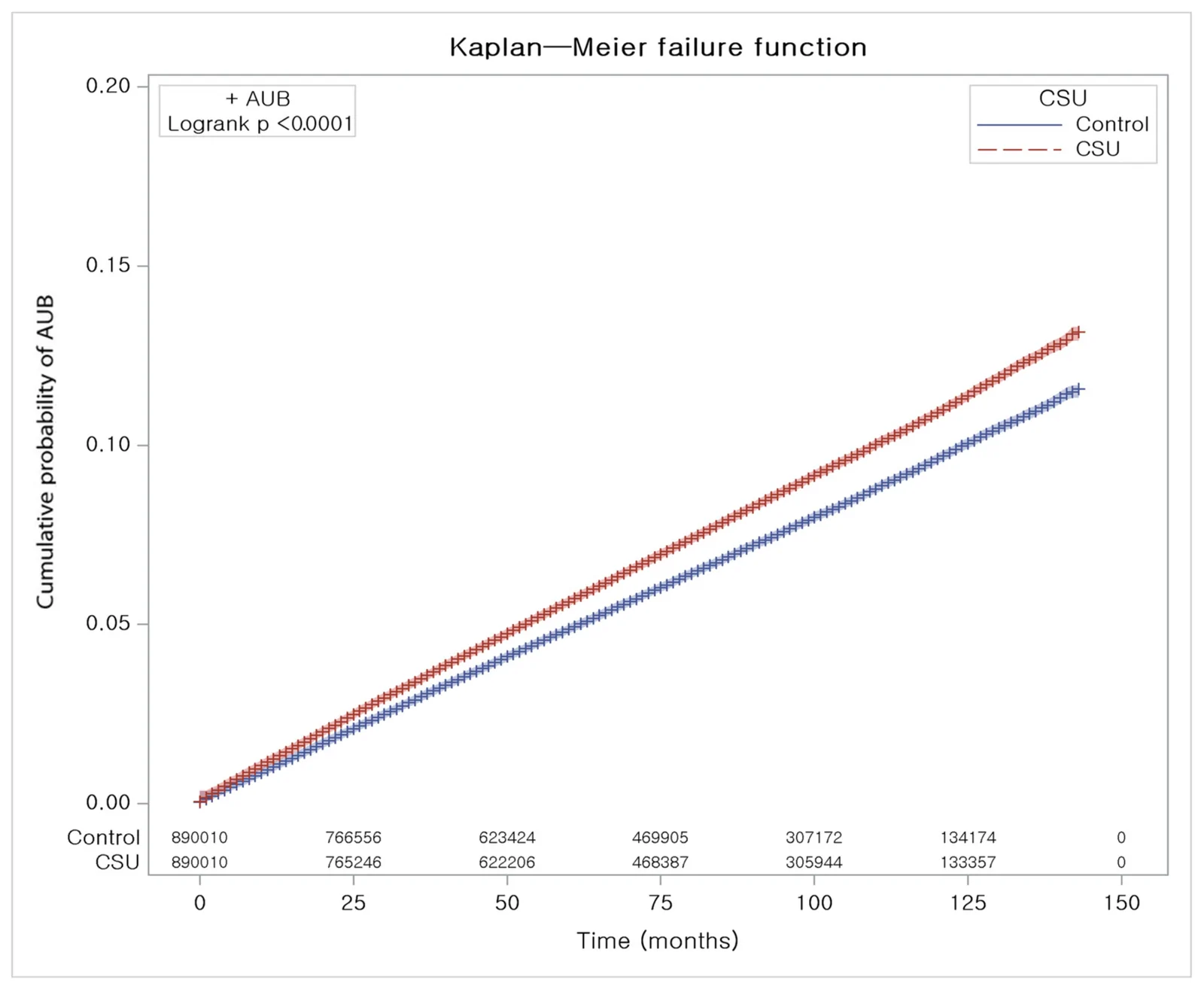
Kaplan–Meier failure curves showing the cumulative probability of abnormal uterine bleeding (AUB) in the chronic spontaneous urticaria (CSU) and matched control groups. The cumulative probability of AUB was higher in the CSU group than in the control group (log-rank *p* < 0.0001). Numbers at risk at the indicated follow-up times are presented below the graph.

Kaplan–Meier failure estimates were used to examine cumulative AUB occurrence in the CSU and control groups (Figure 2). Throughout follow-up, the estimated cumulative probability of AUB remained higher among participants with CSU. The curves differed significantly according to the log-rank test (*p* < 0.001), consistent with the association observed in the Cox models.

## 4. Discussion

Compared with matched controls, participants with CSU were more likely to receive a subsequent diagnosis of AUB. Both Cox proportional hazards regression and Kaplan–Meier analyses supported a modest epidemiological association between the two conditions. However, the magnitude of the association was relatively small (aHR, 1.155; 95% CI, 1.142–1.168). Given the large sample size, the statistical significance of this finding should not necessarily be interpreted as indicating substantial clinical significance. The absolute incidence rate difference of 1.60 cases per 1000 person–years further indicates that the population-level effect was modest.

This study did not directly assess the biological mechanisms underlying the observed association. Nevertheless, hormonal, mast-cell–mediated, and endometrial inflammatory pathways may offer plausible hypotheses for future investigation. Previous studies have suggested that female sex hormones, including estrogen and progesterone, can modulate mast-cell activity [5,6,7]. Progestogen hypersensitivity, which may involve IgE-mediated mechanisms in some susceptible individuals, has also been proposed as a mechanism of enhanced mast-cell reactivity [15]. Kasperska-Zając et al. described a patient whose chronic urticaria occurred concomitantly with an irregular menstrual cycle and improved following oral contraceptive treatment, suggesting a possible relationship between hormonal regulation and urticaria in susceptible individuals [16].

Immune and inflammatory processes also contribute to endometrial breakdown, bleeding, and repair. Berbic and Fraser identified mast cells, macrophages, neutrophils, uterine natural killer cells, and other immune cells as participants in normal menstruation and reported alterations in endometrial immune-cell distribution and inflammatory mediators in women with heavy menstrual bleeding [17]. Mast-cell activation increases as menstruation approaches, and mast-cell- and leukocyte-derived proteases may contribute to extracellular matrix degradation by activating matrix metalloproteinases, thereby participating in endometrial breakdown and bleeding [18]. El-Hamarneh et al. demonstrated increased densities of tryptase-positive, chymase-positive, and c-Kit-positive mast cells in endometrial polyps compared with control endometrium, suggesting localized mast-cell overactivity in an AUB-associated gynecological condition [19]. One possible model is that systemic mast-cell and immune activation in CSU may influence the endometrial immune microenvironment, potentially altering vascular permeability, extracellular matrix remodeling, and local hemostatic and repair processes. Taken together, these observations provide indirect biological plausibility for the hypothesis that hormonal, mast-cell–mediated, and inflammatory pathways may contribute to the relationship between CSU and certain forms of AUB. However, direct evidence demonstrating a shared biological pathway remains limited because these studies did not specifically evaluate endometrial changes in patients with CSU. Prospective studies incorporating sex hormones, inflammatory markers, mast-cell mediators, cytokines, and endometrial biomarkers are needed to evaluate these hypotheses.

Our stratified analysis showed a slightly higher aHR for AUB participants aged <50 years (aHR, 1.159) than those aged 50 and above (aHR, 1.140). The persistence of the association among women aged <50 years is important because this subgroup broadly represents women of reproductive age and is less susceptible to misclassification of postmenopausal bleeding as AUB. Given that hormonal fluctuations are more pronounced in women of reproductive age, this modest difference may be consistent with the hypothesis that hormonal factors contribute to the association between CSU and AUB. However, this possibility requires confirmation because individual menopausal status and hormone levels were unavailable.

These findings complement those of Chen et al., who investigated the risk of CSU among reproductive-aged women with AUB using Taiwan’s National Health Insurance Research Database [20]. In contrast, the present study was specifically designed to evaluate the risk of subsequent AUB among patients with pre-existing CSU by excluding participants with AUB before the index date and identifying newly diagnosed AUB events after CSU diagnosis. Together, these studies suggest a potentially relevant population-level relationship between the two conditions from complementary temporal directions. However, because they were based on separate observational cohorts, neither study establishes causality or confirms a causal bidirectional relationship. Both studies were also conducted in East Asian populations, and no comparable population-based studies directly evaluating this association in other racial or ethnic populations were identified. Therefore, the generalizability of the observed association beyond East Asian populations remains uncertain, and external validation in more diverse populations is needed.

From a clinical perspective, our findings may raise awareness of AUB symptoms among patients with CSU and encourage appropriate gynecological evaluation when clinically indicated. However, given the modest effect size and the lack of evidence demonstrating a clinical benefit from routine screening, the present findings do not support specific screening or treatment recommendations. Nevertheless, greater awareness of AUB symptoms may be warranted in patients with CSU, particularly women of reproductive age, with gynecologic evaluation considered when clinically indicated. Further studies are needed to determine the clinical relevance of this association and to identify whether particular subgroups of patients with CSU may benefit from closer gynecological assessment. These findings also suggest the potential value of collaboration between gynecology and dermatology when managing patients with relevant symptoms.

A major strength of this analysis is the very large cohort drawn from nationwide population data. Exact 1:1 matching for age, income, and residential region improved comparability between participants with CSU and their controls.

Some limitations should be acknowledged. First, CSU and AUB were identified using claims-based operational definitions without individual medical record review. Therefore, we could not directly confirm clinical symptoms, imaging findings, or laboratory results supporting the diagnoses of CSU and AUB. Although we applied exclusion criteria to reduce possible misclassification, residual diagnostic misclassification remains possible.

Second, CSU was identified using physician-assigned ICD codes rather than medical-record review. Although these codes are commonly used in Korean clinical practice for patients considered to have persistent or recurrent urticaria, the NHID did not provide detailed information on symptom duration or eliciting factors. Therefore, we could not directly verify whether wheals and/or angioedema persisted for more than 6 weeks, and no additional criterion requiring repeated diagnoses at least 6 weeks apart or a specified treatment duration was applied. Furthermore, some of the included codes, particularly L50.2–L50.6, may represent inducible forms of urticaria. Consequently, the claims-based operational definition may not correspond exclusively to clinically confirmed CSU, and some degree of exposure misclassification is possible. Future claims-based studies should apply more stringent chronicity criteria and distinguish spontaneous from inducible forms of urticaria.

Third, although the CCI was included to account for differences in overall comorbidity burden, it does not specifically contain several clinical determinants of AUB. Unknown or unmeasured confounding factors, including polycystic ovary syndrome, endometriosis, other gynecological disorders, reproductive history, coagulation disorders, thyroid disorders, hormone replacement therapy, oral contraceptive use, and anticoagulant or antiplatelet therapy, may have influenced the results. Detailed information on parity, menstrual characteristics, individual menopausal status, and the clinical indications for hormone therapy was unavailable because AUB is classified as a sensitive diagnosis in Korean databases, which restricts analyses to customized datasets and prevents the inclusion of medication details. Therefore, despite adjustment for overall comorbidity burden using the CCI, residual confounding by gynecological, reproductive, hematological, and treatment-related factors may remain. As a quantitative sensitivity assessment, the E-value was 1.58 for the observed aHR of 1.155 and 1.54 for the lower confidence limit of 1.142. These values suggest that unmeasured confounding of moderate magnitude could potentially explain the observed association, further supporting cautious interpretation of the findings.

Fourth, women with diagnostic codes indicating menopause or postmenopausal bleeding were excluded; however, individual menopausal status and the timing of menopause could not be confirmed from the claims data. Therefore, some bleeding events among older women may have been misclassified as AUB rather than postmenopausal bleeding. Nevertheless, the association persisted in the age-restricted analysis of women aged <50 years, a subgroup that broadly represents women of reproductive age and is less susceptible to this type of misclassification.

Fifth, AUB was evaluated as a composite outcome encompassing heterogeneous structural and non-structural etiologies. The claims-based diagnostic codes did not allow reliable classification according to the individual PALM-COEIN categories or determination of the cause and severity of each bleeding event. Combining these etiologically distinct conditions may have introduced heterogeneity and diluted a potentially stronger association confined to particular non-structural subtypes, such as ovulatory dysfunction or endometrial disorders. Accordingly, the observed association cannot be attributed to hormonal, inflammatory, or other specific biological mechanisms based on the present data.

Sixth, surveillance bias may also have influenced the observed association. Patients with CSU may have more frequent healthcare encounters, increasing opportunities for AUB symptoms to be detected and coded and potentially inflating the observed association independently of a true biological relationship.

Finally, although participants with an AUB diagnosis before the index date were excluded and incident AUB diagnoses were identified after the diagnosis of CSU, the observational study design cannot establish causality. The findings should therefore be interpreted as an initial population-level epidemiological signal rather than evidence of a causal relationship.

Future research should include detailed gynecologic and imaging findings, laboratory results, and medication data to determine whether the observed association differs among specific causes of AUB. In particular, classification according to the PALM-COEIN system may help determine whether CSU is more strongly associated with specific forms of AUB. Prospective studies and mechanistic investigations are also needed to elucidate the relationship between CSU and AUB and to develop more individualized clinical management and therapeutic strategies. A follow-up study evaluating the incidence of CSU among patients with AUB is also planned and may help determine whether this population-level association is observed in both temporal directions.

## 5. Conclusions

This nationwide cohort analysis showed a modest but significant association between CSU and an increased risk of subsequently diagnosed AUB. The association persisted among women aged <50 years, a subgroup broadly representing women of reproductive age and less susceptible to misclassification of postmenopausal bleeding. Although the present findings do not establish causality or a shared biological mechanism, they provide an initial population-level epidemiological signal supporting further investigation of the relationship between CSU and AUB.

## Figures and Tables

**Table 1:** General Characteristics of Participants.

	CSU (n, %)	Control (n, %)	Standardized Difference
Age			0.000
20–24	4548 (0.51)	4548 (0.51)	
25–29	33,476 (3.76)	33,476 (3.76)	
30–34	58,917 (6.62)	58,917 (6.62)	
35–39	56,030 (6.30)	56,030 (6.30)	
40–44	61,837 (6.95)	61,837 (6.95)	
45–49	91,093 (10.24)	91,093 (10.24)	
50–54	120,021 (13.49)	120,021 (13.49)	
55–59	122,705 (13.79)	122,705 (13.79)	
60–64	106,165 (11.93)	106,165 (11.93)	
65–69	87,491 (9.83)	87,491 (9.83)	
70–74	69,371 (7.79)	69,371 (7.79	
75–79	46,380 (5.21)	46,380 (5.21)	
80+	31,976 (3.59)	31,976 (3.59)	
Income			0.000
1 (lowest)	237,026 (26.63)	237,026 (26.63)	
2	196,850 (22.12)	196,850 (22.12)	
3	133,558 (15.01)	133,558 (15.01)	
4	157,306 (17.67)	157,306 (17.67)	
5 (highest)	165,270 (18.57)	165,270 (18.57)	
Region of residence			0.000
Urban	380,919 (42.80)	380,919 (42.80)	
Rural	509,091 (57.20)	509,091 (57.20)	
Obesity ^†^			0.057
Underweight	39,262 (4.41)	42,979 (4.83)	
Normal	377,121 (42.37)	385,341 (43.30)	
Overweight	203,315 (22.84)	198,139 (22.26)	
Obese I	233,973 (26.29)	226,410 (25.44)	
Obese II	36,339 (4.08)	37,141 (4.17)	
Smoking status			0.064
Non-smoker	847,514 (95.23)	850,887 (95.60)	
Past smoker	16,339 (1.84)	14,848 (1.67)	
Current smoker	26,157 (2.94)	24,275 (2.73)	
Alcohol consumption			0.024
< 1 time a week	676,164 (75.97)	685,128 (76.98)	
≥1 time a week	213,846 (24.03)	204,882 (23.02)	
SBP (Mean, SD)	120.73 (15.46)	121.08 (15.74)	0.023
DBP (Mean, SD)	74.42 (9.82)	74.52 (9.95)	0.011
Total cholesterol (Mean, SD)	197.40 (40.25)	197.49 (39.98)	0.002
Charlson Comorbidity Index (Mean, SD)	0.55 (1.20)	0.52 (1.19)	0.030

^†^ Obesity (BMI, body mass index, kg/m^2^) was categorized as <18.5 (underweight), ≥18.5 to <23 (normal), ≥23 to <25 (overweight), ≥25 to <30 (obese I), and ≥30 (obese II).

**Table 2:** Crude and adjusted hazard ratios (95% confidence interval) of chronic spontaneous urticaria for abnormal uterine bleeding with subgroup analyses according to age, income, and region of residence.

N of Event/ N of Total (%)		Follow-Up Duration (PY)	IR Per 1000 (PY)	IRD (95% CI)	Hazard Ratios for Abnormal Uterine Bleeding			
					Crude ^†^	*p*-Value	Adjusted Model ^†,‡^	*p*-Value
Total participants								
CSU	65,417/890,010 (7.35)	5,221,622	12.50	1.60 (1.54–1.81)	1.154 (1.141–1.167)	<0.001 *	1.155 (1.142–1.168)	<0.001 *
Control	56,819/890,010 (6.38)	5,235,131	10.90		1		1	
Age < 50 years old								
CSU	53,571/425,922 (12.58)	2,686,857	19.90	2.80 (2.57–3.03)	1.163 (1.149–1.178)	<0.001 *	1.159 (1.145–1.174)	<0.001 *
Control	46,560/425,922 (10.93)	2,716,608	17.10		1		1	
Age ≥ 50 years old								
CSU	11,846/464,088 (2.55)	2,534,765	4.67	0.60 (0.48–0.72)	1.150 (1.120–1.181)	<0.001 *	1.140 (1.111–1.171)	<0.001 *
Control	10,259/464,088 (2.21)	2,518,523	4.07		1		1	
Low-income group								
CSU	45,291/567,434 (7.98)	3,369,906	13.40	1.80 (1.67–2.00)	1.167 (1.149–1.185)	<0.001 *	1.161 (1.143–1.179)	<0.001 *
Control	39,258/567,434 (6.92)	3,383,125	11.60		1		1	
High-income group								
CSU	20,126/322,576 (6.24)	1,851,716	10.90	1.42 (1.18–1.59)	1.154 (1.136–1.174)	<0.001 *	1.149 (1.130–1.168)	<0.001 *
Control	17,561/322,576 (5.44)	1,852,006	9.48		1		1	
Urban resident								
CSU	29,479/380,919 (7.74)	2,234,844	13.20	1.90 (1.69–2.10)	1.173 (1.154–1.193)	<0.001 *	1.167 (1.148–1.187)	<0.001 *
Control	25,376/380,919 (6.66)	2,246,365	11.30		1		1	
Rural resident								
CSU	35,938/509,091 (7.06)	2,986,778	12.00	1.50 (1.34–1.68)	1.151 (1.134–1.169)	<0.001 *	1.145 (1.128–1.163)	<0.001 *
Control	31,443/509,091 (6.18)	2,988,766	10.50		1		1	

Abbreviation: IR, incidence rate; IRD, incidence rate difference; PY, person–year. * Stratified cox proportional hazards regression model, Significance at *p* < 0.05. ^†^ Models were stratified by age, income, and region of residence. ^‡^ Adjusted for obesity, smoking, alcohol consumption, SBP, DBP, total cholesterol, and CCI scores.

## Data Availability

The data and materials in this study are available from the corresponding author upon request.

## Abbreviations

The following abbreviations are used in this manuscript:

AUB	Abnormal uterine bleeding
CSU	Chronic spontaneous urticaria
NHID	National health insurance database
SBP	Systolic blood pressure
DBP	Diastolic blood pressure
CCI	Charlson Comorbidity Index
IR	Incidence rate
IRDs	Incidence rate differences
PY	Person–year
CI	Confidence interval
HR	Hazard ratio
aHR	Adjusted hazard ratio
